# Precise Point Positioning on the Reliable Detection of Tropospheric Model Errors

**DOI:** 10.3390/s20061634

**Published:** 2020-03-14

**Authors:** Hongyang Ma, Sandra Verhagen

**Affiliations:** Geoscience and Remote Sensing, Delft University of Technology, 2628CK Delft, The Netherlands; A.A.Verhagen@tudelft.nl

**Keywords:** PPP, tropospheric delay, GNSS, DIA

## Abstract

Precise point positioning (PPP) is one of the well-known applications of Global Navigation Satellite System (GNSS) and provides precise positioning solutions using accurate satellite orbit and clock products. The tropospheric delay due to the neutral atmosphere for microwave signals is one of the main sources of measurement error in PPP. As one component of this delay, the hydrostatic delay is usually compensated by using an empirical correction model. However, how to eliminate the effects of the wet delay during a weather event is a challenge because current troposphere models are not capable of considering the complex atmosphere around the receiver during situations such as typhoons, storms, heavy rainfall, et cetera. Thus, how positioning results can be improved if the residual wet delays are taken into account needs to be investigated . In this contribution, a real-time procedure of recursive detection, identification and adaptation (DIA) is applied to detect the model errors which have the same effects on both phase and code observables; e.g., the model error caused by the tropospheric delay. Once the model errors are identified, additional parameters are added to the functional model to account for the measurement residuals. This approach is evaluated with Global Positioning System (GPS) data during two rainfall events in Darwin, Australia, proving the usefulness of compensated residual slant wet delay for positioning results. Comparisons with the standard approach show that the precision of the up component is improved significantly during the periods of the weather events; for the two case studies, 72.46% and 64.41% improvements of root mean squared error (RMS) resulted, and the precision of the horizontal component obtained by the proposed approach is also improved more than 30% compared to the standard approach. The results also show that the identified model errors are concentrated at the beginning of both heavy rainfall processes when the front causes significant spatial and temporal gradients of the integrated water vapor above the receiver.

## 1. Introduction

The troposphere is the lowest portion of the Earth’s atmosphere, and tropospheric delay due to the neutral atmosphere is one of the main error sources of the Global Navigation Satellite System. This delay can cause up to 2.5 m at zenith direction of the Global Navigation Satellite System (GNSS) signal transmission and over 20 m when satellites are at low elevation angles; e.g., below 10 degree [1,2]. The tropospheric delay is commonly expressed with the following model [3]

(1) $$T\left(e,\alpha \right)=M_{h}\left(e\right)\cdot Z_{h}+M_{w}\left(e\right)\cdot Z_{w}+M_{g}\left(e\right)\cdot cot\left(e\right)\cdot \left(G_{N}\cdot cos\left(\alpha \right)+G_{E}\cdot sin\left(\alpha \right)\right)$$

where *e* and α are respectively the elevation and the azimuth angle of a specific satellite. The total tropospheric slant delay *T* between receiver and satellite at an elevation angle *e* is the sum of three portions: a hydrostatic portion, a wet portion and a gradients portion. $Z_{h}$ and $Z_{w}$ are the zenith hydrostatic delay and zenith wet delay, respectively. $M_{h}$ and $M_{w}$ are the mapping functions for the zenith hydrostatic and wet delay, respectively. $G_{N}$ and $G_{E}$ are the gradients which account for the azimuthally inhomogeneous troposphere in north–south and east–west directions with the corresponding gradient mapping function $M_{g}$.

The hydrostatic delay due to the refractivity of the dry gases in the troposphere can be corrected by the conventional models such as Saastamoinen [1] and Hopfield [4], which can model the hydrostatic delay at the millimeter level in the zenith direction [5]. Collins and Langley [6] proposed a neutral atmosphere model designed for Wide Area Augmentation System (WAAS) users, which is the so-called UNB model series (UNB1 through UNB4) and has been assessed for the use in North America [7,8], Europe [9] and Japan [10]. Li et al. [11,12] developed a multi-dimensional grid model, IGGtrop, to provide tropospheric delay corrections for the users of the BeiDou Navigation Satellite System (BDS) and the area augmentation system based on BDS in China. Although the models mentioned above can correct the wet delay to some extent, the accuracy varies from centimeter to decimeter level, which is still insufficient for high precision positioning and navigation. In addition, using the empirical atmospheric information obtained from the profile of global pressure and temperature may reduce the accuracy of the troposphere models due to the high spatial and temporal variability of water vapor [13,14]. Therefore, the zenith wet delay is usually estimated as an unknown parameter at each epoch or within a certain time span.

When the zenith tropospheric delays are estimated or provided, the slant delays to each visible satellite are obtained by assuming a specific relation between the zenith and slant direction in which the troposphere is assumed to be symmetrical about the vertical direction of the receiver. The relation between zenith and slant delay can be modeled by a so-called mapping function, as already shown in Equation (1), for which a wide range of mapping functions have been developed in the past. The Niell mapping function (NMF) [15] and the global mapping function (GMF) [16] consist of easy-to-handle formulae which only need the input parameters of approximate latitude, height and day of year. On the other hand, the isobaric mapping function (IMF) [17] and the Vienna mapping function 1 (VMF1) [18] provide support for mapping functions derived from numerical weather models (NWM) by applying the ray-tracing technique and/or climatological data. The crucial variable in mapping functions is the elevation angle. Most mapping functions are azimuth-independent, which reveals the underlying assumption that the troposphere is azimuthally homogeneous. A successful application of an azimuthally inhomogeneous tropospheric delay modeling in GPS geodesy and very long baseline interferometry (VLBI) was proposed by MacMillan [19] and Chen and Herring [20], in which the so-called horizontal gradients are considered in addition to a mapping of the zenith to slant delays. In this way, a linear asymmetry of the troposphere is accounted for by introducing a tilted direction instead of the zenith direction. For an extensive review of the troposphere model and mapping function, see Teunissen and Montenbruck [21].

Positioning in severe weather conditions has received more attentions in recent years. Yasyukevich et al. [22] investigated the influence of solar flares on the GNSS and high-frequency propagation. Luo et al. [23] analyzed the performance of double and single-frequency base PPP during three typical geomagnetic storms. As for the tropospheric delay, the standard troposphere model is capable of estimating the tropospheric delay with centimeter accuracy in normal weather conditions [24,25]; however, it should be investigated how positioning results can be improved if the residuals of the tropospheric delay caused by weather events are taken into account. The issue is that satellites at the same elevation angle would be compensated by almost the same tropospheric delay correction based on the standard mapping function approach. However, the symmetrical troposphere about the zenith direction of the receiver is not realistic when it suffers from the complex weather situation. The performance of the horizontal gradients is also limited, because they can only consider a linear asymmetry of the troposphere around the geodetic site [26]. Kleijer [27] analyzed that significant biases can be introduced in the estimated ZWD when the atmosphere is not symmetrical. However, the suggestion of using an accurate wet mapping function is still limited by the assumption of the homogeneous troposphere. Li et al. [28] assessed the impacts of the tropospheric biases on the integer ambiguity resolution and gave the recommendations of under which conditions the tropospheric biases can be ignored. However, only zenith tropospheric biases are taken into account, without considering the biases caused by the inhomogeneous troposphere. Hobiger et al [29] proposed a method to combine the mesoscale and fine-mesh numerical weather model to provide the ray-traced tropospheric slant delay during a typhoon passage. The result shows that the height repeatability is improved up to 30% compared to standard data processing. However, this could still be insufficient for high-precision positioning, and it is not possible to provide the fine-mesh numerical weather model to worldwide users in (near) real-time.

The detection, identification and adaptation (DIA) procedure was first demonstrated by Baarda [30] and Teunissen [31,32]. Teunissen [33] introduced this method into GNSS to detect, identify and adapt the mismodeled errors, and then it was applied in a wide range of GNSS applications; for example, kinematic GNSS surveying [34], permanent station resolution [35] and observation quality control [36,37]. In this contribution, a real-time recursive DIA procedure is implemented to detect the model errors which have the same effects on both phase and code observables, and once the errors are identified, additional parameters will be added to the functional model to account for the measurement residuals. One of the applications of this approach is to detect model errors caused by the tropospheric delay; therefore it was evaluated with GPS data during two different rainfall events in Darwin, Australia, proving the usefulness of compensated residual slant tropospheric delay for positioning results. Comparisons with the standard approach show that the precision of the up component is improved significantly during the period of the weather events, and the precision of the horizontal component is also improved.

This article is organized as follows. Section 2 reviews the standard functional model for PPP data processing and the theory of DIA and the construction of the improved functional model, which takes into account the model errors. Section 3 analyzes the performance of the proposed procedure via two case studies during a weather event. Section 4 contains the summary and conclusions.

## 2. PPP Data Processing

### 2.1. Modeling and Filtering

The undifferenced, ionosphere-free (IF), linear combinations of phase and code are used as the basic observables [38]

(2) $$\begin{array}{cc} \Delta \phi_{r,IF}^{s} & =-{\left(u_{r}^{s}\right)}^{T}\Delta r_{r}+{\mathrm{dt}}_{r}+M_{w}\cdot Z_{w}+M_{g,N}\cdot G_{N}+M_{g,E}\cdot G_{E}+\lambda_{IF}N_{r,IF}^{s}+\varepsilon_{r}^{s}\\ \Delta p_{r,IF}^{s} & =-{\left(u_{r}^{s}\right)}^{T}\Delta r_{r}+{\mathrm{dt}}_{r}+M_{w}\cdot Z_{w}+M_{g,N}\cdot G_{N}+M_{g,E}\cdot G_{E}+e_{r}^{s} \end{array}$$

where $\Delta \phi_{r,IF}^{s}$ and $\Delta p_{r,IF}^{s}$ represent the so-called observed-minus-computed IF combinations for phase and code observable in meters, respectively. Notice that the a priori hydrostatic delay has been corrected for these observations; $u_{r}^{s}$ denotes a unit line of sight vector from satellite *s* to receiver *r*; $\Delta r_{r}$ contains the increments of geodetic coordinate; ${\mathrm{dt}}_{r}$ refers to the receiver clock offset. The satellite clock offset has been corrected by a priori precise products. The wet tropospheric delay $Z_{w}$ is the main interest in this study. The notations of two horizontal gradients are $M_{g,N}:=M_{g}\left(e\right)\cdot cot\left(e\right)\cdot cos\left(\alpha \right)$, $M_{g,E}:=M_{g}\left(e\right)\cdot cot\left(e\right)\cdot sin\left(\alpha \right)$, and the definitions of *e*, α, $G_{N}$ and $G_{E}$ have been illustrated in Equation (1). $\lambda_{IF}$ denotes the wavelength of the IF combination and $N_{r,IF}^{s}$ the IF ambiguity. Note that both receiver and satellite hardware delays have been ignored because they are not the main parameters of interest. $\varepsilon_{r}^{s}$ and $e_{r}^{s}$ are phase and code measurement errors, respectively. It is worth noting that this research only applies the traditional IF combination, and for a more rigorous model, one needs to consider a third observable; that is, the difference between the wide lane phase and the narrow lane pseudorange [39].

After collecting the observed-minus-computed observables $\Delta \phi_{r,j}^{s}$ and $\Delta p_{r,j}^{s}$ at epoch *k*, the IF combination vector of the phase and code $y_{k}$ can be formed from dual-frequency observations. One can symbolize a linear model of the compact formula of Equation (2) as

(3) $$y_{k}=A_{k}x_{k}+e_{k},\;k=1,2,\cdots ,n$$

where $A_{k}$ is the so-called design matrix and $x_{k}$ the n−dimensional state vector containing the unknown estimable parameters; $e_{k}$ refers to the measurement noise vector with $e_{k}∼N\left(0,R_{k}\right)$. The linear dynamic model describing the time evolution of the unknown parameters is given as

(4) $$\begin{array}{cc} x_{k} & =\Phi_{k,k-1}x_{k-1}+d_{k},\;k=2,3,\cdots ,n \end{array}$$

where $x_{k}$ and $x_{k-1}$ refer to the state vectors of the system at epochs *k* and k−1, respectively; $\Phi_{k,k-1}$ represents the transition matrix between two epochs. This matrix is regarded as the identity matrix, because the dynamic system is described by the differential equations of a first-order linearized system, and the identity matrix is obtained by solving the first-order vectorial differential equations. $d_{k}$ represents the system noise at epoch *k* with $d_{k}∼N\left(0,Q_{k}\right)$ and is assumed to be uncorrelated in time.

The initial state of the system and its variance matrix can be given as

(5) $$\begin{array}{cc} {\hat{x}}_{0|0} & ={\left(A_{0}^{T}R_{0}^{-1}A_{0}\right)}^{-1}A_{0}^{T}R_{0}^{-1}y_{0}\\ P_{0|0} & ={\left(A_{0}^{T}R_{0}^{-1}A_{0}\right)}^{-1} \end{array}$$

The time update state vector and its variance matrix are given as

(6) $$\begin{array}{cc} {\hat{x}}_{k|k-1} & =\Phi_{k,k-1}{\hat{x}}_{k-1|k-1}\\ P_{k|k-1} & =\Phi_{k,k-1}P_{k-1|k-1}\Phi_{k,k-1}^{T}+Q_{k},\;k=2,3,\cdots ,n \end{array}$$

where ${\hat{x}}_{k|k-1}$ is the predication of the unknown parameters at epoch *k*, and $P_{k|k-1}$ its corresponding predicted variance matrix.

The predicted residual vector and its variance matrix can be given as

(7) $$\begin{array}{cc} v_{k} & =y_{k}-A_{k}{\hat{x}}_{k|k-1}\\ Q_{v_{k}v_{k}} & =R_{k}+A_{k}P_{k|k-1}A_{k}^{T} \end{array}$$

Using the predicted residual vector, the updated state and its variance matrix are given as

(8) $$\begin{array}{cc} {\hat{x}}_{k|k} & ={\hat{x}}_{k|k-1}+K_{k}v_{k}\\ P_{k|k} & =\left(I_{n}-K_{k}A_{k}\right)P_{k|k-1} \end{array}$$

With the gain matrix

(9) $$K_{k}=P_{k|k-1}A_{k}^{T}Q_{v_{k}v_{k}}^{-1}$$

### 2.2. Detection, Identification and Adaptation

#### 2.2.1. Detection

The objective of the detection step is to detect the mismodeling errors of the mathematical model. The functional model of Equation (2) will be tested at each epoch *k* to detect the presence of model errors; e.g., unmodeled outliers in one or more observations. The distributional property of the predicted error of the unbiased functional model can be expressed as Equation (10) [40,41], which is the so-called null hypothesis model.

(10) $$H_{0}:\;v_{k}∼N\left(0,Q_{v_{k}v_{k}}\right)$$

Otherwise, if the functional model is biased, the distributional property then turns to

(11) $$H_{a}:\;v_{k}∼N\left(C_{v_{k}}\nabla ,Q_{v_{k}v_{k}}\right)$$

where $C_{v_{k}}$ is an m×p matrix with *p* additional unknown parameters and p−vector ∇ is assumed to be unknown. Equation (11) is the so-called alternative hypothesis model.

The test statistic for detecting model errors reads as

(12) $$T_{k}=\frac{v_{k}^{T}Q_{v_{k}v_{k}}^{-1}v_{k}}{r_{k}}$$

where $r_{k}$ is the redundancy at epoch *k*. Equation (12) is also referred to as the local overall model (LOM) test, and model errors are considered to be present at epoch *k* if

(13) $$T_{k}\geq F_{\alpha }\left(r_{k},\infty ,0\right)$$

where $F_{\alpha }\left(r_{k},\infty ,0\right)$ is the critical value based on the central F−distribution with the level of significance α and two degrees of freedom $r_{k}$, *∞*.

#### 2.2.2. Identification

This step is to identify the most likely model error to account for the unexpected effects. For simplification, the case of one single model error at an epoch is considered for each recursion of the DIA process, so the matrix $C_{v_{k}}$ of Equation (11) reduces to a vector, and the test statistic reads as

(14) $$t_{i}=\frac{c_{i}^{T}Q_{v_{k}v_{k}}^{-1}v_{k}}{\sqrt{c_{i}^{T}Q_{v_{k}v_{k}}^{-1}c_{i}}}$$

Commonly, this test is applied to test for outliers in a single observation only. In order to test for an outlier in the *i*th observation, the $c_{i}$-vector should be a 1 as its *i*th element, and zero otherwise. In this study, the goal is to also consider a model error associated with a residual tropospheric delay for one of the satellites due to asymmetry of the troposphere, which implies that the model error has the same influence on both phase and code observables. Thereby, the vector $c_{i}^{T}$ turns to $c_{i}^{T}=\left[0,...,1,...,0,...,1,...,0\right]$ which denotes all the elements in this vector are 0 except for the two 1s corresponding to the ionosphere free combined phase and code observation for one particular satellite. As for the uncombined dual-frequency observable, the *c* vector can extend to four 1s to account for the same model error on two phase observations and two code observations. $v_{k}$ is the predicted residual vector and $Q_{v_{k}v_{k}}$ its corresponding variance matrix; see Equation (7). After computing all the test statistics of the alternative functional models, i.e., for each of the visible satellites, the likelihood of the most likely model error can be determined by comparing the $t_{i}$ with the critical value $N_{\alpha /2}\left(0,1\right)$. For each DIA recursion, among all the satellites with $|t_{i}|\geq N_{\alpha /2}\left(0,1\right)$, the one with the maximum absolute value $|t_{i}|$ is then considered to be the most likely satellite affected by the model errors. After the adaptation step, the DIA process is repeated to test whether additional satellites are affected by model errors.

#### 2.2.3. Adaptation

In case of an outlier, adaption implies disregarding the affected observation. However, in case the model error is affecting both the phase and code observation, it is better to adapt for the error in the functional model as

(15) $$y_{k}^{a}=A_{k}x_{k}+b_{j}\tau_{j}+e_{k}$$

where $b_{j}^{T}={\left[0,...,1,...,0,...,1,...,0\right]}^{T}$; all the elements in this vector are 0 except for the elements of phase and code observation of *j*th satellite is 1, which implies a single additional parameter $\tau_{j}$ is added to account for the unexpected model error of *j*th satellite. In terms of redundancy, this is better than disregarding both observations. However, as explained above, several outliers may occur or several satellites may be affected by a weather event at the same time, and in this case, more than one model error might be identified in a recursive DIA process. Then, the vector $b_{j}$ extends to a matrix *B* and the unknown parameter $\tau_{j}$ extends to an unknown parameter vector τ. The adapted model at *k* epoch then reads as

(16) $$\begin{array}{cc} y_{k}^{a} & =A_{k}x_{k}+B\tau +e_{k}\\ B^{T} & =\left[\begin{array}{c} 0\cdots 1\cdots 0\cdots 0\cdots 0\cdots 1\cdots 0\cdots 0\cdots 0\\ 0\cdots 0\cdots 1\cdots 0\cdots 0\cdots 0\cdots 1\cdots 0\cdots 0\\ \vdots \\ 0\cdots 0\cdots 0\cdots 1\cdots 0\cdots 0\cdots 0\cdots 1\cdots 0 \end{array}\right]\\ \tau^{T} & =\left[\begin{array}{cccc} \tau_{1} & \tau_{2} & \cdots & \tau_{q} \end{array}\right] \end{array}$$

where *B* is an m×q matrix, for which *m* is the dimension of the observation vector and *q* is the number of additional parameters. In each column of *B*, all the elements are 0. except for two 1s, which correspond to the phase and code observation of one satellite. as identified in the recursive DIA procedure.

**Figure 1:**
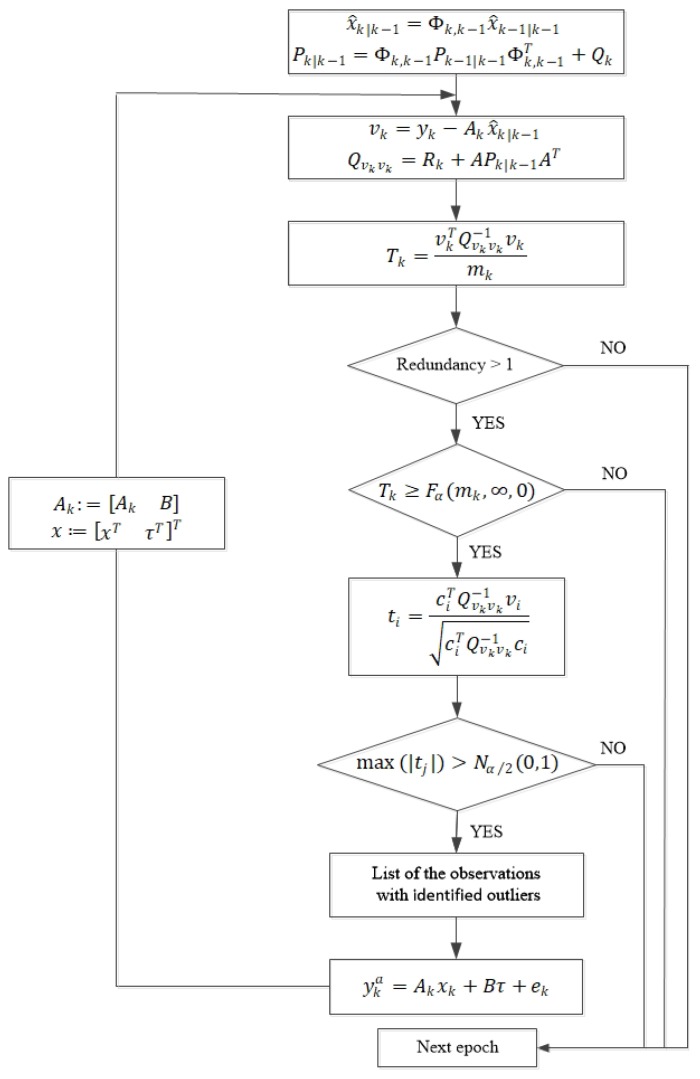
Procedure of detection, identification and adaptation.

The main procedure of this detection, identification and adaptation can be seen in Figure 1

## 3. Case Studies and Results

**Figure 2:**
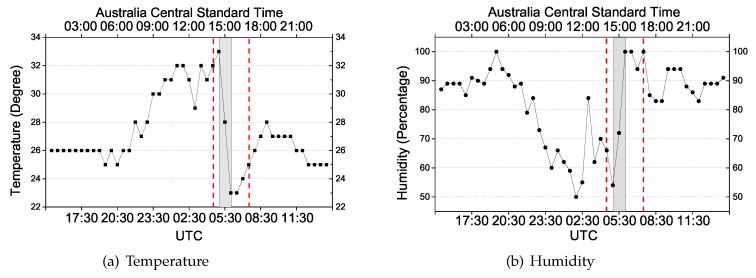
(**a**) Temperature and (**b**) humidity of the corresponding time period of Event 1.

**Figure 3:**
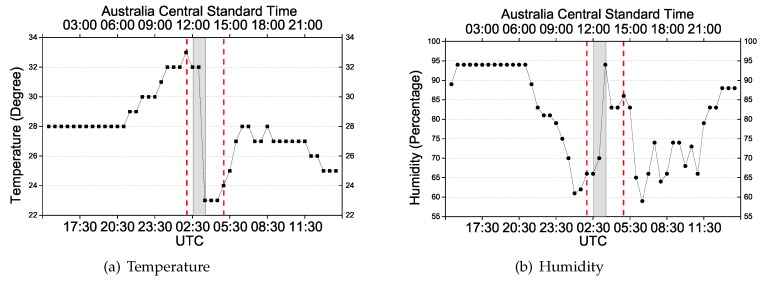
(**a**) Temperature and (**b**) humidity of the corresponding time period of Event 2.

Two case studies will be presented in which we know there was severe weather during the observation period, so as to evaluate the capability of the proposed approach to account for associated model errors due to the asymmetrical behavior of the tropospheric delays during such events. The data of the first event is from the Australian Continuous Operational Reference Station 00NA in Darwin on 14 November 2017. In the sequence, it is referred to as Event 1. The second data set is from 24 March 2018 of an IGS permanent station DARW which is also located in the same region; in the sequence it is referred to as Event 2. In both cases there was heavy rainfall with thunderstorms on the specific days. Temperature and humidity for both days were obtained from https://www.wunderground.com/ and are shown in Figure 2 and Figure 3, respectively. The GPS data and IGS products are used to ensure highly precise orbit and clock corrections. Since this study focuses on the model error detection, the final orbit and clock products are applied in the data processing to eliminate associated errors as much as possible. Configuration of the data processing for the real-time PPP can be seen in Table 1, in which the significance level defines the critical region where the value for test statistic lies in the null hypothesis is rejected.

As for Event 1 of Figure 2, the sun rises at UTC 21:30 (6:00 local time) and then temperature increases while humidity decreases; rainfall appears from 5:00 to 8:00 (UTC) with the temperature dropping 10 degrees within 1 h. A similar effect can also be seen in the change of humidity of that day. For Event 2 of Figure 3, the weather event appears from 2:00 to 5:00 (UTC). The area between the red lines shows the period of the weather event during which a significant influence on the positioning is present. The shadow highlights the period of the dramatic change of temperature and humidity. The hydrostatic delay depends only on the total density of the air, and the change of temperature and humidity would somehow affect the density; thus, with a high probability, the rapid shift in temperature and humidity will impact the hydrostatic delay. However, the inaccuracy of the zenith hydrostatic delay would not be a problem for the proposed model because the residuals of the hydrostatic delay will be lumped into the wet delay. In this case, DIA is to identify the model errors caused by the combined wet delay and residuals of the hydrostatic delay.

**Figure 4:**
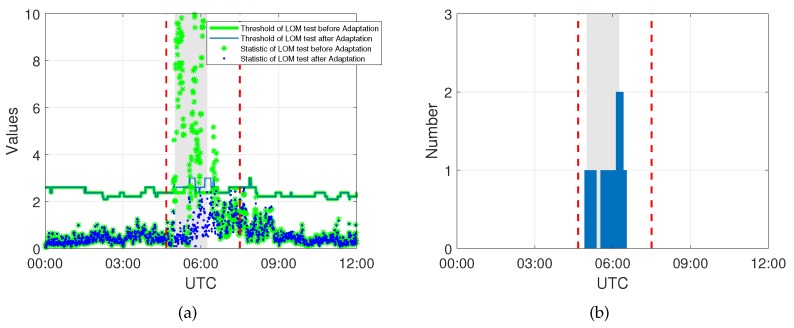
(**a**) Statistics of the Local Overall Model test with the threshold before and after adaptation and (**b**) the model errors identified at each epoch during Event 1.

Figure 4 shows the statistics of the LOM test exceeding the threshold are mostly concentrated at the beginning of the event when the front is passing through, which causes significant spatial and temporal gradients in the integrated water vapor above the receiver. The number of subsequently identified model errors is shown as well, and here, at most two model errors are identified at one epoch, which means only one or two satellites are affected by the event at the same time. Besides, it can be seen that the statistics of the LOM test are below the threshold after the DIA procedure, indicating that there is no indication for remaining undetected model errors.

**Figure 5:**
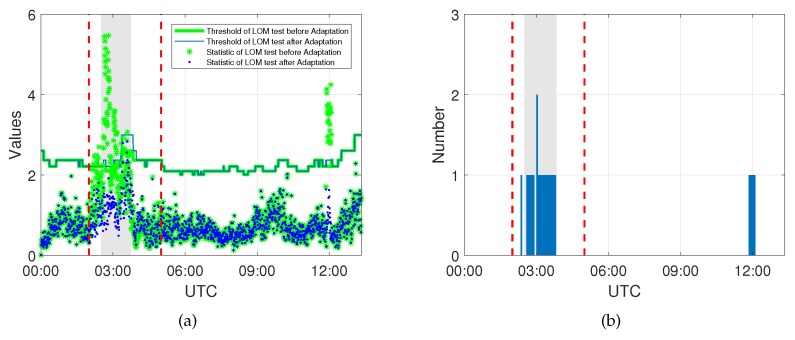
(**a**) Statistics of the Local Overall Model test with the threshold before and after adaptation and (**b**) the model errors identified at each epoch during Event 2.

Similar behavior of the LOM test and identified model errors can also be seen in Figure 5 for Event 2; the rejected LOM test and identified model errors are mostly concentrated at the beginning of the weather event. However, outside the period of this event, there is one satellite detected to be biased at 12:00 UTC. Although it is difficult to prove that these model errors are caused by the tropospheric delay, the results of the up component around 12:00 UTC are also significantly improved, which means the proposed approach is suitable to adapt for model errors which have the same effects on both phase and code measurement.

With the proposed adapted model of Equation (16) during the weather event, the additional parameters which represent the residuals of the slant wet delay are considered to account for the model errors due to the tropospheric delay.

Table 2 and Table 3 are the mean and root mean squared error (RMS) of the phase and code residuals of satellites being identified with the model error during the weather events of Event 1 and Event 2, respectively. As can be seen in these two tables, residuals of the phase observations of the affected satellites are mostly reduced because the adjusted functional model is more reliable with the additional parameters accounting for the model errors. As for the residuals, the improvement of the phase observation is more significant than that of the code observation, because the value of the additional parameter mainly depends on the phase observation due to its much higher weight compared to the code observation.

**Figure 6:**
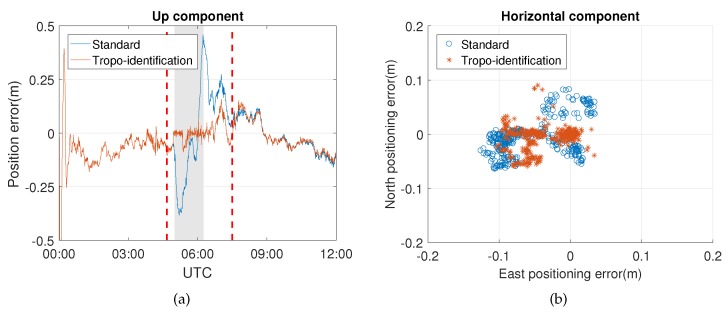
(**a**) Position errors of the up component and (**b**) the horizontal component of Event 1.

**Figure 7:**
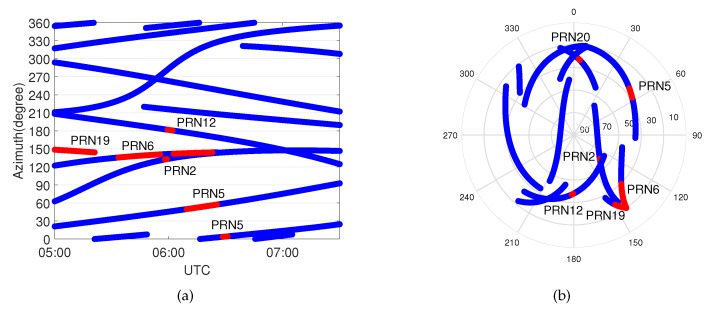
Occurrences of model errors (indicated in red) during Event 1 as function of (**a**) time and azimuth and (**b**) elevation and azimuth (skyplot). The blue lines represent the trajectories of the satellites, and the red points indicate that model errors are identified at those epochs.

Figure 6 illustrates the results of the up component and the horizontal component within the time span from 5:00 to 8:00 UTC of Event 1. The pattern of the up component positioning error with the standard approach represented as a blue line shows a typical trend affected by the tropospheric delay. On the contrary, the vertical positioning errors with the proposed method are reduced because the residual slant wet delays have been compensated by the additional parameters. Although there is still a systematic error in the east direction, the performance of the proposed method in horizontal displacement is better than that with the standard approach during the weather event. As can be seen in the skyplot of Figure 7, most of the affected satellites are located in the east part of the skyplot, which leads to a partially biased horizontal component after adjusting.

Table 4 summarizes the improvement of the results obtained from the proposed method compared to the standard approach. Significant improvement can be seen in the up component, since it is known that the tropospheric delay is one of the main error sources in the vertical direction due to high correlation. The horizontal precision of the proposed method is also improved by about 30%. For Event 2, most of the satellites affected by the weather event are located in the east part of the skyplot (Figure 9).

The distribution of the influenced satellites is shown in the skyplot of Figure 7.

**Figure 8:**
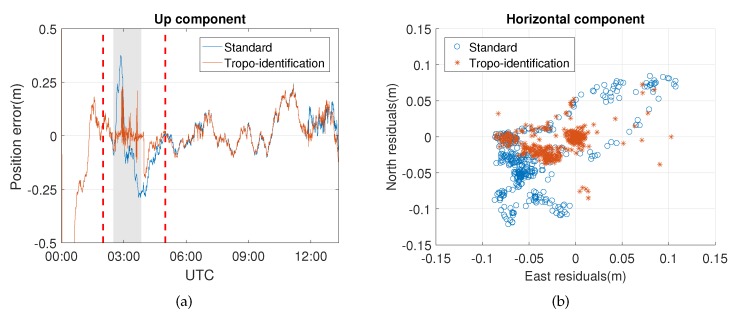
(**a**) Residuals of the up component and (**b**) the horizontal component during Event 2.

**Figure 9:**
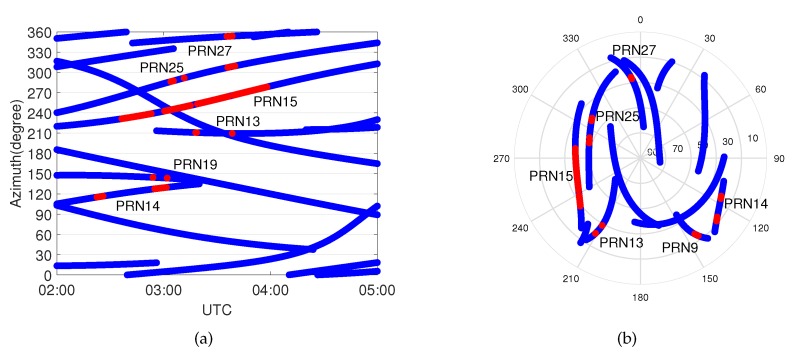
Occurrences of the model errors (indicated in red) during Event 2 as function of (**a**) time and azimuth and (**b**) elevation and azimuth (skyplot). The blue lines represent the trajectories of the satellites, and the red points indicate that model errors are identified at those epochs.

Similarly, the positioning errors of the up component of Event 2 in Figure 8 are also reduced, since the effects of the weather event have been removed. This approach also works well for the aforementioned model errors identified outside the period of the weather event at around 12:00 UTC, indicating that the model errors can be compensated if they have the same influence on the phase and code observables. Table 5 shows a significant improvement of the up component, which is the same as Event 1. Meanwhile, a system error still exists in the east component, though the precision of the horizontal component is also improved. From the skyplot of Figure 9, one can see that most of the influenced satellites are concentrated in the west part of the site, which partly causes the east-west bias of the horizontal component.

Occurrences of the model’s errors during Event 1 are shown in Figure 7 as functions of time and azimuth, and elevation and azimuth, respectively. The blue lines show the trajectories of the satellites, and the red points indicate at which epochs a model error was identified.Most of the identified model errors are concentrated within the azimuth angle range 60∼150 degrees; i.e., the east part of the skyplot. At the beginning of the weather event, two satellites, PRN19 and PRN6 at around 150 degrees, were affected by the event. Both of them are at low elevation angles, as shown in the skyplot of Figure 7. When PRN2 and PRN12 approached this area, they also identified with model errors, which means signals are affected by an extra tropospheric delay in this direction compared to any other azimuth angles. Then, the front moved from 150 degrees to 60 degrees, and thus satellite PRN5 and PRN20 were affected, after which it dissipated. It is worth noting that wrong identification might be present; e.g., PRN6 is affected by the weather event for a long period of time and among which PRN2 at almost the same azimuth angle as PRN6 is identified with the model error for several epochs.

Figure 9 shows the distribution of the identified model errors in Event 2, which resembles the distribution of Event 1 in which the model errors are concentrated within a certain range of azimuth angle; i.e., ≈210–300 degrees. This property may indicate the heading direction of the front. The event keeps affecting PRN15 for a long time, which is not at a low elevation angle. Satellites close to this range of azimuth angle are also detected with model errors at some epochs.

**Figure 10:**
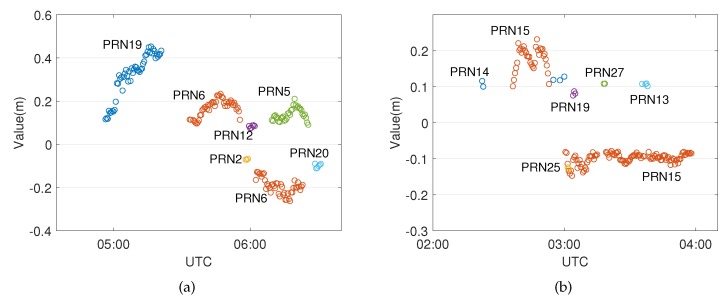
Values of the estimated additional parameters of (**a**) Event 1 and (**b**) Event 2.

Figure 10 shows values of the estimated additional parameters which are due to the unmodeled slant wet delays caused by the weather events for this specific case. For Event 1 on the left side, the model error of PRN19 reaches up to more than 40 cm, as this satellite goes down to a low elevation angle. It seems reasonable, since the wet delay may lead to a delay of up to several meters at a low elevation angle. The mean and RMS phase residuals of PRN19 shown in Table 2 reduce, respectively, to −0.01 mm and 0.04 mm when compensated by the estimated additional parameters. The negative values of the additional parameters are because of the mismodeled hydrostatic delay. The mean and RMS phase residual of PRN6 also drop to 0.31 mm and 7.3 mm, respectively.

As for Event 2, values of PRN15 change rapidly from +20 cm to –10 cm, and they are stable for a long time span; even these additional parameters are considered to be epoch independent. This implies a further implementation of the global overall model test which takes into account the test statistics over a period of time rather than a single epoch. As can be seen in Table 3, the mean and RMS phase residuals of PRN15 reduce to –2.24 mm and 14.73 mm, respectively, which still show significant improvements compared to the standard PPP without the DIA procedure.

## 4. Conclusion

In this contribution, a DIA procedure was implemented to identify the model errors which have the same impact on both the phase and code observables; one of the applications is to account for model errors caused by tropospheric delays. An improved functional model was proposed with the additional parameters to account for the model errors. Although precise orbit and clock products were applied in the data testing to avoid any other model errors, this troposphere identification model can be easily implemented in real-time PPP, and the DIA procedure can be processed in real-time. This procedure was evaluated by two case studies of weather events, during which the tropospheric delay might be azimuthal asymmetric around the receiver, and thus the model errors due to the inhomogeneous troposphere can be detected by this procedure. The phase residuals of the satellites identified with model errors are compared to the standard approach during the weather events, since the unmodeled wet delay can at least be partly absorbed in the additional parameters. The positioning results are also improved during the events, and the improvement is most significant for the up component (72.46% and 64.41% improvement of RMS for two weather events) since the tropospheric delay is one of the main error sources in the vertical direction. The positioning performance of the horizontal component obtained from the proposed method is also improved (more than 30% improvement of RMS) compared to the standard PPP. The values of the additional parameters indicate the model errors due to the tropospheric delay can reach 40 cm when the satellite is at a low elevation angle.

At most two model errors are identified at one epoch in the two case studies, which indicates that not too many satellites are affected by the asymmetrical troposphere, even during the weather events. Despite the complexity of extreme weather, the identified model errors are concentrated at the beginning of both heavy rainfall processes when the front causes significant spatial and temporal gradients of the integrated water vapor above the receiver. Besides, the satellites affected by the events are concentrated within a certain range of azimuth angle, which is related to the path of the front. This proposed procedure can also be used in monitoring severe weather. If the outliers detected by this method increase dramatically, it may indicate the front line of weather event is passing through. More testing shows that the proposed procedure may not always bring a very large improvement, but as least it does not deteriorate the positioning solutions; meanwhile, it does prevent severe impacts in some cases.

## Figures and Tables

**Table 1:** Configuration of the data processing for the real-time PPP.

Items	Values
STD of the zenith wet delay	0.2 m [42]
Process noise of the zenith wet delay	0.02 m/$\sqrt{h}$
STD of the gradients	0.01 m
Process noise of the gradients	0.001 m/$\sqrt{h}$
Interval 30 s
STD of phase	0.005 m [43]
STD of code	0.5 m
Significance level	0.005

**Table 2:** Means and RMSs of the phase and code residuals of the satellites being identified with model error during Event 1. The unit is mm. The abbreviations Sta and Pro represent the standard and proposed PPP approaches, respectively, and Ipv represents the improvement of the proposed approach compared to the standard approach.

PRN	Phase						Code					
	Mean			RMS			Mean			RMS		
	Sta	Pro	Ipv(%)	Sta	Pro	Ipv(%)	Sta	Pro	Ipv(%)	Sta	Pro	Ipv(%)
2	−5.44	−3.40	37.50	13.11	11.09	15.41	−334.78	−324.93	2.94	729.88	724.16	0.78
5	7.10	1.33	81.27	21.22	12.73	40.01	−109.28	−72.45	33.70	568.05	574.76	−1.18
6	−5.26	−0.31	94.11	33.89	7.30	78.46	11.78	8.22	30.22	418.58	404.25	3.42
12	6.21	3.43	44.77	21.88	9.73	55.53	−117.01	97.61	16.58	346.31	339.23	2.04
19	2.43	−0.01	99.59	16.72	0.04	99.76	74.60	71.33	4.38	362.67	360.11	0.71
20	−3.37	2.17	35.61	24.62	11.69	52.52	−334.59	−312.85	6.50	627.26	615.74	1.84

**Table 3:** Means and RMSs of the phase and code residuals of the satellites being identified with model error during Event 2.

PRN	Phase						Code					
	Mean			RMS			Mean			RMS		
	Sta	Pro	Ipv(%)	Sta	Pro	Ipv(%)	Sta	Pro	Ipv(%)	Sta	Pro	Ipv(%)
13	5.93	5.11	13.83	19.82	15.72	20.69	−166.57	−167.91	−0.80	416.06	418.79	−0.66
14	5.16	5.18	−0.39	22.79	20.30	10.93	133.27	131.68	1.19	467.72	467.22	0.11
15	−7.63	−2.24	70.64	32.50	14.73	54.68	−615.33	−618.36	−0.49	719.20	721.64	−0.34
19	−2.69	−2.45	8.92	13.30	15.40	−15.79	−41.41	−41.47	−0.14	331.28	330.85	0.13
25	7.72	1.24	83.94	24.11	16.30	32.39	−497.78	−500.91	−0.63	593.69	596.46	−0.47
27	−7.29	−5.51	24.42	18.20	14.86	18.35	620.12	622.21	−0.34	954.67	954.27	0.04

**Table 4:** Statistics of the mean and RMS residuals for PPP with and without DIA during Event 1. The unit is *m*.

	Mean			RMS		
	Sta	Pro	Improve	Sta	Pro	Improve
Up	0.046	0.021	54.35%	0.207	0.057	72.46%
East	−0.054	−0.035	35.19%	0.071	0.048	32.39%
North	−0.006	−0.006	0%	0.034	0.023	32.35%
3D	0.198	0.063	68.18	0.222	0.079	64.41%

**Table 5:** Statistics of the mean and RMS residuals for standard approach and proposed method during Event 2. The unit is *m*.

	Mean			RMS		
	Sta	Pro	Improve	Sta	Pro	Improve
Up	−0.053	−0.010	81.13%	0.161	0.062	61.49%
East	−0.038	−0.021	44.74%	0.059	0.037	37.29%
North	−0.027	−0.007	74.07%	0.056	0.020	64.29%
3D	0.159	0.057	64.15	0.181	0.075	58.56%
